# Modern Methods in Crown and Bridge Work

**Published:** 1901-07-15

**Authors:** J. L. Young

**Affiliations:** Detroit, Mich.


### MODERN METHODS IN CROWN AND BRIDGE WORK.

BY DR. J. L. YOUNG, DETROIT, MICH.

Read before the Tri-State Meeting, Indianapolis, Ind., June 4—6, 1901.

The following is a brief outline: The basis of all crown work, especially such as is intended to support bridge work, in the collar or band around the tooth or the stump of the root. As a rule all bands should pass beneath the gum at least one thirty-second part of an inch, whether for porcelain or gold crowns, and should not project above the gum enough to show an excessive amount of the metal used, especially where used on the anterior teeth.

The upper cuspid teeth are often employed for the attachment of one or more missing adjacent teeth, and as these teeth are often free from caries, conscientious operators hesitate to cut off the crowns of these teeth to make bridge attachments and hence resort to what is known as the open-face collar. Because of the peculiar shape of the cuspid teeth, being either short and thick, or long with nearly equilateral proximal surfaces, or very much constricted necks, it is not always possible to adopt an open-faced crown to these teeth so that it will not loosen and permit recurrance of caries under the bands. It is also impracticable to preserve the interproximal spaces if the bands are made sufficiently heavy to withstand the strain of more than a one-tooth bridge. Cements used in the attachment of such bands is exposed to the dissolving influences of the saliva to such an extent as to very materially shorten the life of the bridge.

It is preferable generally to sacrifice the pulps of good teeth so that a firm and permanent anchorage may be secured for the attachment of bridges. It is the author's conviction that a more useful structure will result and the life of all teeth or roots involved will be prolonged.

The author's method of removing crowns and pulps, when necessary, is to incise such crowns as can be so treated and inject the pulp with sterile cocain solution while still paralyzed from the shock of incision. Then remove the anesthetized pulp and fill root at once, using every precaution to prevent infection. For teeth that can not be so treated, the occlusal surfaces are ground down with corborundum stones until sensitive and then anesthetized by cataphoresis and the pulp removed as before.

The shaping of molar stumps for cap crowns is done after removal of the pulp by grinding the proximal and lingual and buccal surfaces to the desired extent with corborundum disks and stones and then rounding the corners with diamond disks which have been swadged saucer shape. The shaping of the stump should be of such extent as to permit desirable contouring to preserve interproximal spaces.

As additional support the essayist advocated using saddles where long spaces were to be bridged; that is, a narrow plate swadged to fit the gum and upon which the bridge is to be constructed. These are particularly desirable in porcelain bridge work, as the bridges need not be made so bulky to get desired strength.

The essayist described his method of constructing a porcelain bridge from the upper cuspid to first molar on the same side; also, one in which the Mason facings were employed.

The essayist maintained that the essential features of good bridge work were first, good and secure anchorage; and secondly, strong and substantial construction, using metal of such thickness that it will not change shape under the strain of mastication; thirdly, solder of high grade should always be used and limited in quantity to an amount which will equalize the construction in every part. Solders of low caret and used in excessive quantities contract unduly and distort the several elements in cooling so that easy setting of the bridge is not possible.

DISCUSSION.

Dr. J. Stephan, of Cleveland, Ohio: Every one has his pet methods. I shall not intrude mine at this time. Can't agree with the essayist in condemning all open-face bands, because I don't care to mutilate well-formed, sound teeth when they can be used without mutilation, and safely, too. Neither do I agree with him in using seamless crowns as bridge attachments. It is not essential that a band pass beneath the gum in every case, but if it does not pass beneath it should not closely approach it; there must be a self-cleansing space, or one that can be readily cleansed by the patient.

In making open-faced collars I sometimes dress the proximal surface considerably to allow of ready insertion of the bridge without distorting it, but in every case the ground surface must be covered by the metal of the collar and the cement used to set the collar. I am also very particular to bevel and finish nicely the edges of metal in the interproximal space and where it laps over onto the tooth, just the same as though it were a filling. This renders cleansing easy and does not invite accumulations of putrefactive substances.

I don't like the heroic method of incising teeth and extirpating pulps; I prefer cataphoresis or the cocain pressure method.

I prefer to make bridges strong enough so that they need not rest on the gums at all. I find that saddle bridges are difficult to keep clean, and in time produce a diseased condition of the soft membranes on which they rest.

My method of making bicuspid dummies is to grind facing to position and then grind the occlusal end to a forty-five degree miter, and adapt to its flat surface a platinum backing by swedging it between two pieces of semi-hard rubber. To the mitered occlusal end is then fitted the swedged cusps and waxed to place. The porcelain face is then removed and solder flowed into the metal back and cusps uniting them and contouring and finishing to some extent. The pin holes in the backing are then drilled out and the porcelain facing returned to its place and the dummy assembled with the other parts of the bridge and invested for soldering, which is done with a solder fusing at a lower temperature than that used to make the bulk of the dummy.

Dr. N. S. Hoff, of Ann Arbor, Mich.: The essayist condemns all use of the open-faced crown, and, it seems to me, without sufficient reason. These crowns, when imperfectly fitted or unsubstantially made, are unquestionably to be condemned. They should not either be placed in the mouth of a patient who will not take care of his teeth in a reasonable manner. There may be some cuspid teeth to which it is impossible to fit an open-face crown, but there are few which may not, by slight changes which will not mutilate the tooth, be made more serviceable by this method than by the entire removal of the crown and its substitution with an artificial one. No artificial crown can be made as artistic, and the chances are that it will not be as durable.

Dr. C. V. Vignes, of New Orleans, La.: I have abandoned all open-faced crowns, because they do not stand more than two or three years. The cement washes out and the bridge loosens, or they furnish spaces for the accumulation of putrefactive material which causes destruction of the entire tooth by a gradual solution of its hard tissues.

In making soldered dummies I do not leave the lingual surfaces uncontoured. My method is to grind porcelain facing to place and back with gold and platinum plate, then form the cusps and attach to backing and facing with adhesive wax, built out to full contour of the tooth to be represented. Then on the proximal sides apply No. 60 gold and platinum foil, leaving the lingual face with only the wax showing. Then invest and boil out the wax, leaving a metallic box or matrix into which flow solder until full. This will produce a solid contoured crown or dummy which may be assembled and soldered into the bridge. Its advantages are that it is easily and quickly made and is at the same time artistic.

Dr. W. A. Price, of Cleveland, O.: We should not condemn the pulps in teeth where they can be saved, not even to secure foundations for bridge work. I say this because I think it is impracticable to properly remove the pulp from one-half the canals and to securely fill them so that detrimental results may not sooner or later take place. I don't like to replace the natural crowns with porcelain crowns, especially in early life, as the teeth naturally change color as the patient gets older, and a porcelain crown that is eight or ten years old will not be in harmony with its neighbors, necessitating the removal of the bridge and a change.

